# Readability and Comprehension of Printed Patient Education Materials

**DOI:** 10.3389/fpubh.2021.725840

**Published:** 2021-11-30

**Authors:** Pálma Szabó, Éva Bíró, Karolina Kósa

**Affiliations:** ^1^Faculty of Medicine, Department of Behavioral Sciences, University of Debrecen, Debrecen, Hungary; ^2^Doctoral School of Health Sciences, University of Debrecen, Debrecen, Hungary; ^3^Faculty of Medicine, Department of Public Health and Epidemiology, University of Debrecen, Debrecen, Hungary

**Keywords:** health literacy, comprehension, patient education, healthcare workers, readability index

## Abstract

**Background:** Health literacy, a recently determined construct plays an important role in how individuals are able to manage their health. A useful approach for the assessment of health literacy is to measure the comprehension of available patient education materials (PEMs).

**Objective:** We aimed at assessing the usefulness of PEMS available in Hungarian by testing comprehension of selected PEMs in different groups of users.

**Methods:** Comprehension of patient education materials in the domain of healthcare was tested by selecting PEMs and creating questions based on their text in 3 dimensions of health literacy: understand, process/appraise, apply/use. Twenty questions were created that could be answered without pre-existing knowledge by reading the appropriate text taken from PEMs. Comprehension was examined in four groups: laypersons, non-professional healthcare workers, 1st year healthcare students, and 5th year medical students. Readability indices were calculated for the same texts to which questions were created.

**Results:** Laypersons answered <50% of the PEMs-based questions correctly. Non-professional healthcare workers performed better with 57% of right answers but significantly worse than healthcare students or medical students. Those with at least high school qualification (maturity exam) showed significantly higher comprehension compared to those with lower educational attainment. Persons in good or very good health also had significantly better comprehension than those in less favorable health. All readability indices showed that comprehension of the tested PEMs required at least 10 years of schooling or more. Therefore, these PEMS are difficult to understand for persons with less than high school level of education.

**Conclusion:** Rephrasing of the investigated patient educational materials would be recommended so that they better fit the educational attainment of the Hungarian population. Evaluation of the readability and comprehensibility of other PEMs also seems warranted.

## Introduction

According to an early definition of the term, health literacy is the degree to which individuals have the capacity to obtain, process and understand basic health information and services to make appropriate health decisions (1). A more recent definition of Sorensen and the HLS-EU Consortium (2) based on a systematic literature review proposed a more complex definition according to which health literacy “entails people's knowledge, motivation and competences to access, understand, appraise, and apply health information in order to make judgments and take decisions in everyday life concerning healthcare, disease prevention and health promotion to maintain or improve quality of life during the life course.” An integrated model, built on this wider definition identifies at least four dimensions of health literacy in three health domains: health care, disease prevention and health promotion.

Health literacy is assessed at the individual or population level using one or more of the large numbers of validated instruments (3, 4). International surveys found that sizable proportions of the populations in developed countries had less than sufficient levels of health literacy. For example, 36% of the adult US population had below-basic or basic level of health literacy in 2003 (5), and the proportion of persons with inadequate or problematic health literacy ranged from 28.7% in the Netherlands to 62.1% in Bulgaria in the European health literacy survey (HLS-EU) (6).

An obvious aim is to improve the level of health literacy. Until then, helping people comprehend health-related information can be achieved by creating easy-to-understand materials (7). One step in this process is to assess the comprehension and readability of existing written patient education materials (PEMs) (8, 9) since these are routinely used in health care and have been shown to improve self-management of various conditions (10, 11). In case of comprehension, understanding of relevant material by individuals is tested (12). Readability of a text is assessed by calculating various readability indices based on formulas that use the number of syllables or characters in a specific text. Indices reflect the difficulty of the vocabulary and sentences in written materials and can be assigned to a “grade level” to express the number of years of schooling which would be required to comprehend the given text.

The most frequently used readability indices are the Flesch-Kincaid Index (FKI), the Gunning-Fog Index (GFI), the Simple Measure of Gobbledygook (SMOG) (13, 14), and the Coleman-Liau index (CLI) (15). The former three are calculated using the number of syllables, words, and sentences in a text which are fed into a specific weighted formula to produce a total score in a range that corresponds to a particular US school level. The formula for calculating CLI uses the number of characters in a text instead of syllables. These readability indices are primarily used for English texts. However, the Flesch Reading Ease Test from which the FKI index is calculated, the Gunning-Fog Index, and the Simple Measure of Gobbledygook were also tested and found useful in Hungarian texts (16). The CLI has also been used for languages other than English and can be used for comparing the readability of various texts in the same language, with higher numbers reflecting more difficult texts (17).

The suggested reading level for PEMs are grade 6–8. However, the readability scores of several existing PEMs seem to be significantly higher than that in the UK, Canada and Australia (18, 19).

Readability assessments according to various indices have been carried out on English PEMs for patients with chronic kidney disease (20), dermatological diseases (9), and PEMs available at the point of care (21). There were similar studies carried out on PEMs for patients at menopause (22), with congestive heart failure (23), as well as on PEMs for orthopedic or rheumatology patients (19, 24), also for patients undergoing hand surgery (25) and for various other common health conditions (26, 27).

A recent paper even addressed readability for patient education material on COVID-19 (28).

Our goal was to assess the usefulness of patient education materials by a two-pronged approach, investigating both comprehension and readability. PEMs used in the Hungarian health care system were collected in the most important areas of patient-doctor interactions: scheduling an appointment, giving consent, scheduling and side-effects of medication, side-effects of surgical procedure, dietary recommendations, finding health care services, health insurance-related and ethical guidelines. Comprehension of these texts was investigated by creating questions based on the texts. Readability of the same texts was assessed by calculating four indices (FKI, GFI, SMOG and LKI).

Comprehension of PEMs was assessed in laypersons and non-professional health workers of primary health care. These workers had no professional qualification and were employed as health mediators in a large-scale model programme that was designed to introduce group practices (so-called GP clusters) in the primary care system of Hungary. These group practices also offered previously unavailable preventive services such as health status assessment, lifestyle counseling, and community health promotion programmes in regions with sizable numbers of disadvantaged patients. Non-professional workers acted as facilitators between professional workers and the serviced population with the aim of easing communication, increasing access and uptake of health services, and aiding health promotion programmes (29). Patient education was not a specific task for health mediators but they were frequently asked to read and interpret PEMs by patients in the community, so comprehension of these texts was a salient question.

## Materials and Methods

### Selection of Patient Education Materials

Considering the large number of PEMs used in the Hungarian health care, we decided to limit the study to those in the domain of health care as defined by Sorensen et al. (2). Of the four dimensions in this model, the first (“accessing/obtaining information relevant to health”) was omitted since this was not relevant in the present study. PEMs were selected that covered major issues of health care in the other three domains in which patients have to understand and process information and make decisions. Only patient education materials produced and distributed by the largest health care provider of the North-Eastern region of the country were selected since lay persons and patients in the target groups would most frequently receive these materials. Texts from PEMs were selected to cover the most important issues in each of the 3 dimensions as shown in Table 1.

**Table 1:** Topics of the patient education materials selected for assessing comprehension.

**Domain: Health care**	**Dimensions of health literacy investigated in the present study**		
	**Understand information relevant to health**	**Process/appraise information relevant to health**	**Apply/use information relevant to health**
Issues	4. Consent form—analyzing complications 5. Insurance claim 6. Insurance claim 11. Obtaining imaging results 17. General prognosis of a chronic disease 18. Prognosis of chronic disease in a specific case	3. Potential of complication based on Consent form 8. Dietary recommendations 9. Laboratory results 10. Laboratory results 15. Organ donation law 19. Side effects of medication 20. Side effects of medication	1. Medication regimen 2. Medication regimen 7. Dietary recommendations 12. Opening hours of a pharmacy 13. Opening hours of a pharmacy 14. Medication regimen 16. Scheduling appointment for checkup
Number of questions	6	7	7

### Creation of Questions for Testing Comprehension

Selected PEMs were reviewed and texts of no more than one paragraph with information describing conditions or situations relevant to issues in one of the 3 investigated dimensions (Table 1) were identified. Questions were formulated based on the text of PEMs so that all questions could be unequivocally answered—without pre-existing knowledge—by reading and comprehending the preceding text. Each question had one right answer and at least but no more than 3 potential other (wrong) answers (altogether 2, 3 or 4 answers) to choose from. Twenty questions were formulated in 12 topics from 12 PEMs. Pilot testing was carried out by health professionals with at least 5 years of work experience who found the texts and corresponding questions to be clear and answerable, not requiring adjustment. The created questionnaire is referred to as Competency in Patient Care (CPC).

### Sample and Data Collection for Testing Comprehension

Non-professional workers (health mediators) employed in the model programme were invited to participate (*n* = 35). Lay participants of a community health promoting programme were also asked to participate (*n* = 130). Data collection took place in May-June 2016. In order to compare the performance of lay persons and non-professional workers, 1st year students of physiotherapy and dietetics (*n* = 54) and medical students in their final year of education (*n* = 29) were invited to read the same texts and answer the same questions. Data collection in the latter two groups was carried out in December 2018-February 2019.

### Evaluation of the Test of Comprehension

The number of right answers was calculated for each respondent for all items. The proportion of right answers from all respondents was calculated for each item. The number of potential answers for each item varied between 2 and 4. This resulted in different probabilities of chance to find the right answer for each item which was taken into account by correction in the following way. The percent of correct responses for each question in each occupational group was divided by the probability of chance given the actual number of potential answers for each question. For example, if the right answer had to be chosen from 2 answers, the probability of finding it by chance was 50%; if the right answer had to be chosen from 4 answers, the random probability of finding the right one was 25%.

### Statistical Analysis

**Figure 1:**
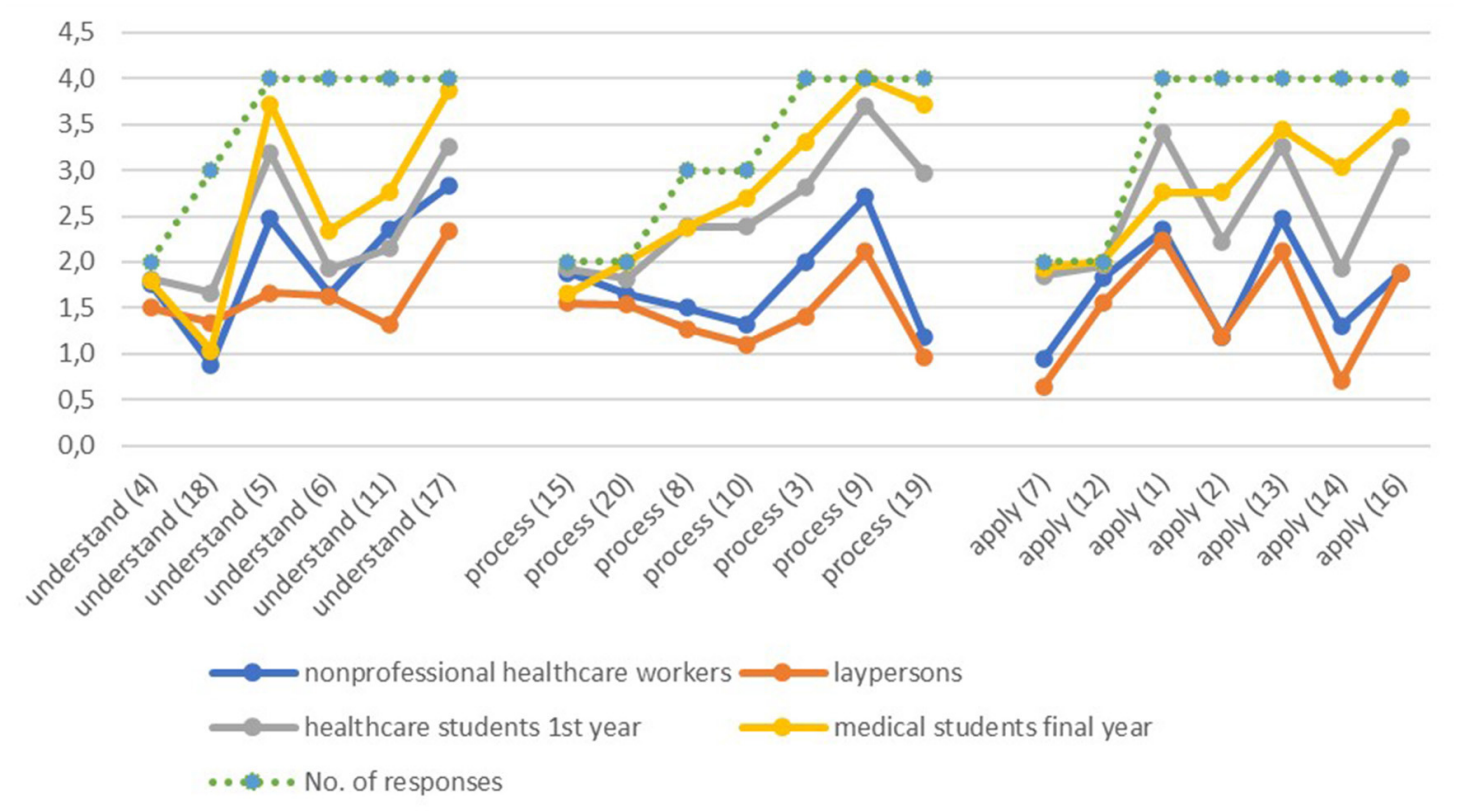
Distribution of the corrected proportion of right answers by dimensions of health literacy by occupational groups. Green dots show the total number of responses (2, 3, or 4) on each item.

The proportion of right answers was calculated. The uncorrected proportion of right answers is shown in Table 2, and with correction (for the probability of choosing an answer randomly) in Figure 1 where the “number of responses” means the total number of responses for each question from which the right answer had to be chosen. The proportion of right answers is corrected accordingly. Out of 20 questions, 5 questions had 2 potential answers, 3 questions had 3 potential answers, and 12 questions had 4 answers from which the one right answer had to be selected. Comparison of the proportion of right answers in the various groups was carried out by the Kruskal-Wallis test. Calculations were carried out in MS 365 Excel and Stata 16.1.

**Table 2:** Features of the participants by occupational/study group.

	**Non-professional health workers**	**Laypersons**	**Students of physiotherapy and dietetics**	**Medical students**
N	34	125	54	29
Age (mean ± SD, years)	missing	37 years (±14.91)	21 years (± 1.61)	24 years (±1.11)
Sex (%, males)	15	10	6	28
Highest level of education				
Primary % (*N*)	18 (6)	46 (57)	0	0
Secondary % (*N*)	82 (28)	47 (58)	0	0
In progress (university students) or completed tertiary % (*N*)	0	7 (9)	100 (54)	100 (29)
Marital status				
Single % (*N*)	12 (4)	27 (34)	100 (54)	100 (29)
Present partnership (married/cohabiting) % (*N*)	68 (23)	49 (61)	0	0
Former partnership (divorced/widowed) % (*N*)	20 (7)	24 (30)	0	0
Subjective health status				
Very good/good % (*N*)	53 (18)	49 (61)	85 (29)	69 (11)
Fair % (*N*)	41 (14)	40 (49)	15 (5)	31 (5)
Bad/very bad % (*N*)	6 (2)	11 (14)	0	0

### Assessment of Readability

Four measures of readability were calculated for the total text of the test of comprehension. Three of those indices (Flesch Kincaid Index, Gunning-Fog Index, Simple Measure of Gobbledygook) are based on the number of syllables in a text; the fourth (Coleman-Liau index) is based on the number of characters.

To calculate the Flesch Reading Ease test, the total number of sentences, words and syllables were counted in the texts and fed into the Flesch formula to calculate the score as follows: Flesch Reading Ease score = 206.835—(1.015 × ASL)—(84.6 x ASW) where ASL is the total word count divided by the total sentence count; ASW is the total syllable count divided by the total word count (30). Result of the Flesch Reading Ease Test can be converted to the Flesch-Kincaid Index which specifies the grade level of the text.

The Gunning-Fog Index is calculated as follows: 0.4 × [(total word count/total sentence count) + 100 × (number of complex words (3 or more syllables)/total word count)] (31, 32).

The SMOG Index was described by McLaughlin (33): 3+ **√** complex words per 30 sentences (34).

The Coleman-Liau index has the following formula: CLI = 0.0588L−0.296S−15.8 where L is the average number of letters per 100 words, S is the average number of sentences per 100 words (15).

A web-based tool was used to calculate all indices (35). This calculator analyzes the grade reading level of English text using a series of readability indices, including the ones listed above. The text was cleaned beforehand, that is, periods marking the end of each heading, sentence fragment, or sentence were removed.

## Results

### Assessment of Comprehension

Two hundred and forty-eight participants returned the questionnaire of which 6 were excluded from evaluation because more than 50% of answers were left blank. Demographic features of the 242 respondents included in the study are shown in Table 2.

Without correction for the random choice of right answers, the mean comprehension of each item ranged between 33.47 and 86.78%. The mean proportions of right answers by item and dimension are listed in each occupational category in Table 3. The overall proportion of right answers was significantly different by occupational groups: 56.7% among non-professional health workers, 47.6% in laypersons, 76.7% in students of physiotherapy and dietetics, and 82.7% among medical students (*p* < 0.01).

**Table 3:** Uncorrected proportion of right responses by item and occupational/study groups.

	**Non-professional health workers**	**Lay-persons**	**Students of physiotherapy and dietetics**	**Medical students**
Understand information (%)				
4. Potential complications of a surgical procedure based on the consent form	88.2	75.2	90.7	89.7
5. Insurance claim after mild accident	61.8	41.6	79.6	93.1
6. Insurance claim after severe accident	41.2	40.8	48.2	58.6
11. How to request imaging results	58.8	32.8	53.7	69.0
17. General prognosis of a chronic disease	70.6	58.4	81.5	96.6
18. Prognosis of chronic disease in a specific case	29.4	44.8	55.6	34.5
Process/appraise information (%)				
3. Identification of potential complications in a consent form	50.0	35.2	70.4	82.8
8. Calculation of dietary intake in diabetic diet	50.0	42.4	79.6	79.3
9. Identification of abnormal laboratory results	67.7	52.8	92.6	100
10. Impact of food consumption on laboratory results	44.1	36.8	79.6	89.7
15. Interpretation of the law on organ donation in a specific case	94.1	77.6	96.3	82.8
19. Identification of potential side effects of a specific medicine	29.4	24.0	74.1	93.1
20. Symptom as a potential side effect of a specific medicine	82.4	76.8	90.7	100
Apply/use information (%)				
1. Application of a specific medicine by age	58.8	56.0	85.2	69.0
2. Application of a specific medicine in children	29.4	29.6	55.6	69.0
7. Food choice in low-fat diet	47.1	32.0	92.6	96.6
12. Which pharmacy is open now	91.2	77.6	98.2	100
13. Which pharmacy will be open in a specific future timepoint	61.8	52.8	81.5	86.2
14. Can a specific medicine be halved	32.4	17.6	48.2	75.9
16. Choosing a date for checkup based on specific information	47.1	47.2	81.5	89.7

We also analyzed the correct number of answers taking into account the varying number of potential responses (between 2 and 4) from which the correct answer had to be selected as described in Methods. This way the proportion of right answers was corrected by the probability of choosing an answer randomly: the proportion became lower in case of a higher number (>2) of potential answers compared to when the right answer had to be selected only from 2 potential answers.

The corrected proportions of right answers are shown by each item and occupational group in Figure 1. Green dots show the potential number of responses on each item. This corrected evaluation shows even more clearly the difference between the occupational groups. The figure also reveals questions which can be considered good or easy—the ones which most respondents answered correctly (4, 12, 15). The most difficult questions (6, 18) had a low proportion of correct answers even by medical and healthcare students. These related to the interpretation of insurance claim and organ donation law. Questions 5, 17, 10, 19, 16 had the highest differentiating power which were mostly correctly answered by healthcare and medical students, and mostly incorrectly by lay persons and non-professional health mediators.

We analyzed overall comprehension, that is, the proportion of right answers by gender, education, occupational group, and health status by the Kruskal-Wallis test as described in Methods. Results are summarized in Table 4. Except for gender, significantly different comprehension was found among subgroups of other variables. Those with at least maturity exam gave 23.5% more correct answers compared to those without (*p* < 0.001); medical and healthcare students selected 27.6% more correct answers compared to laypersons and non-professional healthcare workers (*p* < 0.001); and those in at least good subjective health gave 13.4% more right answers than those in adequate or worse health (*p* < 0.001).

**Table 4:** Comprehension in the subgroups by socio-demographic variables and subjective health.

	**Per cent of all right answers**	***p***
**By gender**		
Male (*N* = 28)	58.93	0.820
Female (*N* = 213)	59.86	
**By education**		
No maturity exam (*N* = 102)	46.03	<0.001
Maturity exam (*N* = 140)	69.50	
**By marital status**		
Single (*N* = 121)	69.66	<0.001
Present partnership (*N* = 84) (married or cohabiting)	48.27	
Former partnership (*N* = 37) (divorced or widowed)	52.43	
**By occupation**		
Laypersons	47.60	<0.001
Non-professional health workers	56.76	
Students of physiotherapy and dietetics	76.76	
Medical students	82.76	
**By subjective health status**		
Good/very good (*N* = 153)	64.54	<0.001
Fair/bad/very bad (*N* = 89)	51.12	

### Assessment of Readability in Comparison With Available Health Literacy Tools

Readability indices such as the FKI, CLI, SMOG and GFI were calculated for our test of comprehension (Competency in Patient Care, CPC), and also for the Hungarian versions of some widely used tests of health literacy. CPC was found to be at 12th grade level by the Coleman-Liau Index, at 11th grade level by FKI (10.6), at 10th grade level by the SMOG index (9.8), and at 13th grade by GFI (13.2 for GFI is defined as ‘hard to read’). The readability indices of the widely used health literacy tools such as NVS, HLS-EU 47, BRIEF and S-TOFHLA were also calculated and compared to CPC. The readability indices of CPC are similar to the readability indices of validated health literacy tools, all requiring at least 10 years of education (Table 5).

**Table 5:** Comparison of readability scores of the assessed health literacy tools.

	**CPC**	**NVS**	**S-TOFHLA**	**BRIEF**	**HLS-EU 47**
FKI	10.6	10.6	12.7	12.4	12.7
Gunning-Fog	13.2	12.9	15.3	14.3	13.5
SMOG	9.8	9.5	11.6	10.3	9.9
CLI	12.0	11.0	8.0	11.6	19.0

## Discussion

Our study tested the comprehension of patient education materials in various occupational groups, among them non-professional health workers who are supposed to help lay people access and use health care services and understand health-related information. Overall comprehension of the investigated PEMs among laypersons was around chance, that is, their comprehension was no different from selecting answers randomly, as opposed to answers based on the provided information. Comprehension among non-professional health workers was slightly better than chance and was considerably worse than that of students of medical and health care professions. Comprehension of the latter two groups was adequate. However, medical students in their final years performed way below expectations in terms of one issue, and their performance was only slightly better than chance in 3 more issues, all of them related to comprehension of insurance claims and ethical issues.

We also tested the readability of the same materials used for comprehension testing by calculating the most frequently used readability indices such as the Flesch-Kincaid Index (FKI), Gunning-Fog Index (GFI), Simple Measure of Gobbledygook (SMOG) as their usability was previously shown for Hungarian texts (16).

Both the test of comprehension and the readability indices suggest that the language of PEMs is not tailored properly to the wide range of potential users in the Hungarian population. Considering that 45.87% of the 15–74 year-old population had no high school diploma (no maturity exam), and 21.21% of the population had only primary education or less in 2018 according to the Hungarian Central Statistical Office (36), the investigated PEMs seem to be too difficult for those with no maturity exam.

Readability indices (FKI, GFI, SMOG) previously used for Hungarian texts were also calculated for the text of the test of comprehension, and their scores also suggest that the language of PEMs is certainly not tailored properly to the population with lower educational attainment than high school diploma. The CLI had a much wider range being way below (S-TOFHLA) or way above (HLS-EU 47) other indices of the same questionnaire so its interpretation requires caution.

Readability indices do not necessarily reflect whether a given material is effective since they only focus on individual words and sentences, and do not take into account the active role of the reader. Therefore, these indices do not measure comprehension, and indices for the same text may differ in their grade level assignment (27). However, since they can be used to measure any text for any purpose, they can be useful as a first approach to assess patient education materials and compare the grade level of different versions of the same material.

Our results are in concert with earlier findings of the American Medical Association according to which most health care materials are written at a 10th grade level or higher although most adults read between the eighth and ninth grade level (37).

Since increasing numbers of patients use an increasing number of digital educational resources, the creation of clear and effective PEMs is more important than ever. Guidelines have been available for the creation of easy-to-understand health messages and patient education materials for more than a decade (7, 38). Their general recommendation is to write as simply as possible without sacrificing content or distorting meaning. However, this seems to be a tall order as the readability assessment of a number of PEMs attest (21, 39, 40). The readability of PEMs aimed at patients with various conditions has been found to exceed that of recommended levels. Comprehension of topics involving legal matters such as insurance and medical ethics seem to be difficult even for medically qualified professionals as was shown by our questionnaire.

One of the limitations of our study is that it gives information about the readability of the selected PEMs based on text only. Charts, tables and images cannot be evaluated. Furthermore, the readability formulas were originally validated for English texts, though some of these scores were previously used with Hungarian texts (16).

Comprehension of the PEMs measured during our study does not provide in-depth information about health literacy though it can raise concerns regarding the required skills to understand, appraise and apply health information in healthcare, disease prevention and health promotion.

The strength of our study is its novelty to assess the readability of Hungarian PEMs and to reveal a gap between the recommended and the actual level of readability of such materials. Our findings underline the need for a review of patient education materials in use and evaluation of new materials before release along with the health literacy of patients who are supposed to use them. Difficulties or incomprehension of patient education materials is a grave problem since people cannot act upon information they do not comprehend. In optimal cases, patient education materials should not only be easy to comprehend but should also be tailored to the specific characteristics of the intended target group (41).

## Conclusions

There seems to be a large discrepancy between the readability of the educational materials and the reading level of the general population. Considering that people with lower educational attainment are at higher risk for morbidity and mortality compared to those with higher levels of schooling, the previous group has been in a much greater need of clear health communication using plain language than the latter. More extensive research should be conducted to evaluate the readability and comprehensibility of available PEMs. In addition, rephrasing of existing education materials using simple language seems necessary, or even establishment of an organization responsible for editing such information materials as it is exemplified in Canada (42).

## Data Availability Statement

The raw data supporting the conclusions of this article will be made available by the authors, without undue reservation.

## Ethics Statement

The studies involving human participants were reviewed and approved by the Model Programme had been implemented in the framework of the Swiss Contribution Programme SH/8/1 that specified the indicators for evaluation. Indicators monitored in primary health care are specified in the Hungarian Health Care Act of 1997 (1997. évi CLIV. törvény az egészségügyrol). The Programme was created on the basis of the Framework Agreement between Switzerland and Hungary (declared by 348/2007. (XII. 20.)) and signed on 20 December 2007. Ethical approval for data collection for research purposes in the Programme was issued by the Scientific and Research Ethics Committee of the Medical Research Council of Hungary (ETT-TUKEB) (16676-3/2016/EKU (0361-16). The patients/participants provided their written informed consent to participate in this study.

## Author Contributions

PS collected PEMs, created the questionnaire for comprehension testing, collected data, performed statistical analysis, and drafted the manuscript. ÉB contributed to the creation of questionnaire, data collection, and drafting of the manuscript. KK designed the study, supervised creation of the questionnaire for comprehension testing, supervised statistical analysis, drafted the manuscript, and approved its final version. All authors have read and agreed to the published version of the manuscript.

## Funding

Part of the study was carried out in the framework of the Public Health Focused Model Programme for Organizing Primary Care Services Backed by a Virtual Care Service Center. The Model Programme was implemented in the framework of the Swiss Contribution Programme SH/8/1 that had been supported by a grant from Switzerland through the Swiss Contribution to Hungary. The authors were supported during the writing of the manuscript by the GINOP-2.3.2-15-2016-00005 project financed by the European Union under the European Social Fund and European Regional Development Fund. The funders had no influence on study design, data collection and analyses, interpretation of results, writing of the manuscript or the decision to submit it for publication.

## Conflict of Interest

The authors declare that the research was conducted in the absence of any commercial or financial relationships that could be construed as a potential conflict of interest.

## Publisher's Note

All claims expressed in this article are solely those of the authors and do not necessarily represent those of their affiliated organizations, or those of the publisher, the editors and the reviewers. Any product that may be evaluated in this article, or claim that may be made by its manufacturer, is not guaranteed or endorsed by the publisher.

## Abbreviations

BRIEF: Brief Health Literacy Screening Tool
CLI: Coleman-Liau index
CPC: Competency in Patient Care
FKI: Flesch-Kincaid Index
GFI: Gunning-Fog Index
HLS-EU: European Health Literacy Survey
HLS-EU 47: European Health Literacy Survey Questionnaire 47
NVS: Newest Vital Sign
PEMs: patient education materials
SMOG: Simple Measure of Gobbledygook
S-TOFHLA: Short-Test of Functional Health Literacy in Adults.
